# The ecological and physiological bases of variation in the phenology of gonad growth in an urban and desert songbird

**DOI:** 10.1016/j.ygcen.2016.03.013

**Published:** 2016-05-01

**Authors:** Scott Davies, Samuel Lane, Simone L. Meddle, Kazuyoshi Tsutsui, Pierre Deviche

**Affiliations:** a School of Life Sciences, Arizona State University, Tempe, AZ 85287, USA; b The Roslin Institute & Royal (Dick) School of Veterinary Studies, University of Edinburgh, Roslin, Midlothian EH25 9PS, UK; c Laboratory of Integrative Brain Sciences, Department of Biology and Center for Medical Life Science, Waseda University, Tokyo 162-8480, Japan

**Keywords:** Gonad cycles, Food abundance, Phenotypic plasticity, Reproductive neuroendocrinology, Urbanization

## Abstract

Birds often adjust to urban areas by advancing the timing (phenology) of vernal gonad growth. However, the ecological and physiological bases of this adjustment are unclear. We tested whether the habitat-related disparity in gonad growth phenology of male Abert’s towhees, *Melozone aberti*, is due to greater food availability in urban areas of Phoenix, Arizona USA or, alternatively, a habitat-related difference in the phenology of key food types. To better understand the physiological mechanism underlying variation in gonad growth phenology, we compared the activity of the reproductive system at all levels of hypothalamo-pituitary–gonadal (HPG) axis. We found no habitat-associated difference in food availability (ground arthropod biomass), but, in contrast to the seasonal growth of leaves on desert trees, the leaf foliage of urban trees was already developed at the beginning of our study. Multiple estimates of energetic status did not significantly differ between the non-urban and urban towhees during three years that differed in the habitat-related disparity in gonad growth and winter precipitation levels. Thus, our results provide no support for the hypothesis that greater food abundance in urban areas of Phoenix drives the habitat-related disparity in gonad growth phenology in Abert’s towhees. By contrast, they suggest that differences in the predictability and magnitude of change in food availability between urban and desert areas of Phoenix contribute to the observed habitat-related disparity in gonad growth. Endocrine responsiveness of the gonads may contribute to this phenomenon as desert – but not urban – towhees had a marked plasma testosterone response to GnRH challenge.

## Highlights

• We found no habitat-associated difference in food availability (arthropod biomass).
• Unlike desert areas, urban areas had no detectable seasonal change in tree growth.
• Desert, but not urban birds, had a marked plasma T response to GnRH challenge.
• The timing of reproductive development varied inter-annually only in desert birds.

## 1 Introduction

Phenotypic plasticity, the ability of an organism to alter its phenotype in response to environmental change, is a crucial adaptation to seasonal environments (Lourdais et al., 2002, Miner et al., 2005, Wingfield and Kenagy, 1986). A major seasonal phenotypic change for many animals is the cycle of gonad growth (Bubenik et al., 1997, Dawson, 1983, Itoh et al., 1990), which is regulated by a rise in reproductive hormone secretion (Deviche et al., 2010, Wingfield and Kenagy, 1986). It is often assumed that inter-individual and inter-annual variation in the phenology of hormone secretion determines corresponding variation in the timing of gonad growth. However, within a given population, large intra-individual differences in plasma levels of reproductive hormones at a given time mean that they rarely reflect fine-scale (i.e., less than a month) differences in gonad phenology (Caro et al., 2006, Davies et al., 2015a, Schaper et al., 2012a, Schaper et al., 2012b, Williams, 2008). Therefore, the mechanism underlying inter-individual and inter-annual variation in the phenology of gonad growth is currently unclear.

In most vertebrates, gonad growth results from an endocrine cascade initiated by neuroendocrine responses to environmental cues (Dawson, 2008, Palmer et al., 1988, Wingfield, 2008). In birds, the endocrine activity of the hypothalamo-pituitary–gonadal (HPG) axis is stimulated by a photoinduced increase in gonadotropin-releasing hormone 1 secretion (GnRH-1; Sharp, 2005). This neuropeptide stimulates the secretion of luteinizing hormone (LH) from the anterior pituitary gland, which is crucial for gonad growth and gametogenesis (Kuenzel, 2000). In males, the testes secrete testosterone (T), which is essential for spermatogenesis (Dornas et al., 2008, Penfold et al., 2001). Gonad growth is also modulated by another hypothalamic neuropeptide, gonadotropin-inhibitory hormone (GnIH; Tsutsui et al., 2000), which opposes the actions of GnRH-1 (Clarke and Parkington, 2013, Tsutsui et al., 2013). Specifically, in birds GnIH inhibits gonad growth by acting on hypothalamic GnRH-1 cells, anterior pituitary gland gonadotropes, and gonads (Tsutsui et al., 2013). GnIH also has links with hypothalamic cells that produce neuropeptide Y (NPY; Boswell, 2001), a neuropeptide that integrates information on food availability into endocrine activity of the HPG axis (Marty et al., 2007). For seasonally breeding birds, within the window of reproductive development set by photoperiod, these processes are fine-tuned to local environmental conditions by supplementary cues, such as ambient temperature (Caro et al., 2013, Schaper et al., 2012b), social factors (Maney et al., 2007), food abundance (Davies and Deviche, 2014, Hahn et al., 2005, Perfito et al., 2008), and/or the phenology of particular food types (Visser et al., 2006, Watts and Hahn, 2012).

Birds are one of the best studied taxa in urban ecology and the available evidence suggests that one adjustment of birds to persist in urban areas is an advancement of the phenology of seasonal gonad growth, relative to their non-urban conspecifics (Deviche et al., 2010, Deviche and Davies, 2014, Partecke et al., 2005). Accordingly, our multi-year study of male Abert’s towhees, *Melozone aberti*, found that this songbird advanced vernal gonad growth phenology in urban areas of Phoenix, Arizona compared to desert areas (Davies et al., 2015a). This advancement was mirrored by advancement in the phenology of the vernal increase in plasma LH, but not plasma T. To better understand the physiological control of gonad cycles in urban and desert towhees, we tested two non-mutually exclusive hypotheses. The first hypothesis was that the advanced phenology of gonad development of towhees in Phoenix arises at the brain level. If this is the case, we predicted that the phenology of gonad growth would be related to the amount of hypothalamic neuropeptides that ultimately modulate gonad growth (i.e., GnRH-1 and GnIH). The second hypothesis was that the disparity in gonad growth between towhees in Phoenix versus desert areas is due to differences in the endocrine responsiveness of the anterior pituitary gland and/or the gonads. For this, we exposed birds to a GnRH challenge, which involved measuring the increase in plasma LH and T in response to exogenous GnRH (Deviche et al., 2012a, Jawor et al., 2006). This technique has the potential to be insightful in terms of the physiological control of seasonal gonad growth because it minimizes intra-individual variation in hormone levels (McGlothlin et al., 2010), is individually repeatable (Jawor et al., 2007), and the birds’ responsiveness to GnRH challenge varies as a function of their breeding stage (Jawor et al., 2006) and energetic status (Davies et al., 2015b).

The ecological driver of the advanced gonad growth phenology of urban birds is likewise unclear. Urbanization has the potential to modify many supplementary environmental cues used by birds to time gonad growth. A particularly compelling candidate environmental factor that is both modified by urbanization and plays an important role in gonad growth phenology in birds is food availability (reviewed by Deviche and Davies, 2014). Food availability potentially influences gonad growth phenology via two distinct scenarios (Davies and Deviche, 2014, Deviche and Davies, 2014, Hahn et al., 2005). In the first, the overall abundance of food modulates the energetic status of birds. Accordingly, birds in good energetic condition can begin gonad growth shortly after stimulation by sufficient photoperiod, whereas birds in poor energetic condition delay gonad growth until they have acquired sufficient energy stores. In Phoenix, urbanization is associated with increased arthropod abundance (Cook and Faeth, 2006). Increased arthropod abundance increases food abundance for towhees, which feed predominantly on ground arthropods. Thus, it is predicted that urban towhees will be in better energetic status and can begin gonad development as early as photoperiod allows. The second scenario by which food availability may influence gonad growth phenology posits that the phenology of seasonally available key food types, such as growing green vegetation and arthropods, acts as an environmental cue forecasting the occurrence of optimal conditions (Davies and Deviche, 2014, Deviche and Davies, 2014, Hahn et al., 2005). Crucially, this scenario is hypothesized to operate via perceptual pathways independent of energetics (Hau et al., 2000). The growing season of plants in urban areas of Phoenix begins earlier than that of desert areas (Buyantuyev and Wu, 2010), which likely advances the availability of key nutritional resources. Hence, the second aim of this study was to test the hypothesis that food abundance and the phenology of plant growth differ between urban and desert areas of Phoenix. We predicted that the timing of gonad growth would be related to the phenology of food abundance (ground arthropod dry biomass) and the phenology of leaf foliage progression in urban and desert locations.

## 2 Methods

### 2.1 Study sites and species

Our study was conducted at three desert and four urban sites (Davies et al., 2013). For a map and quantitative comparison of the study sites, see Davies et al. (2015a). All procedures were approved by the Arizona State University Institutional Animal Care and Use Committee and conducted under appropriate scientific collecting permits issued by the Arizona Game and Fish Department and the US Fish and Wildlife Service.

### 2.2 Bird capture, blood collection, and morphometrics

Between 14th March and 5th April 2013, we caught adult male Abert’s towhees using a mist net and song playback (Tweit and Finch, 1994). We caught birds between 06:28 AM and 10:13 AM (mean capture time: urban = 07:49 AM; desert = 07:53 AM). There was no difference in the date of capture between treatment groups in either study (see below) or when both studies were combined (*P*’s ⩾ 0.58).

The stress of capture causes a rapid decrease in plasma T in many vertebrate species, including multiple species of birds (Deviche et al., 2012b). Therefore, we bled all birds within 3 min of capture to quantify pre-stress (hereafter referred to as ‘initial’) hormone levels. All blood samples (∼200 μl) were collected from the right jugular vein using a heparinized syringe, and were immediately placed on ice until centrifuged (within 6 h) and the plasma harvested. Plasma samples were then frozen at −80 °C until they were assayed. We also measured body mass (±0.5 g, using a Pesola spring balance), wing chord (±0.5 mm), and cloacal protuberance (CP) width (±0.1 mm, using digital calipers). Body mass and wing chord were linearly related (linear regression: *r*^2^ = 0.42, *P* = 0.003), so we used these parameters to calculate body condition (as scaled mass index, Peig and Green, 2009). We also quantified fat stores by assigning a score of 0–5 (0 representing no fat, 5 representing bulging fat deposits) to the amount of fat visible in the furcular region.

### 2.3 Plant and arthropod phenology

To examine whether plant phenology differed between the two habitats, we measured leaf foliage progression from three desert and four urban localities every three weeks. To directly compare the two habitats, we scored three tree species that were common to both habitats: mesquite (*Prosopis* spp.), palo verde (*Parkinsonia* spp.), and salt cedar (*Tamarix* spp.). Measurements were taken from one 100 meter transect at each locality and we scored all trees within 10 m either side of each transect. Leaf progression score represented a qualitative six-point scale of foliage progression (1 = no leaves or leaf buds, 2 = buds closed, 3 = buds splitting slightly, 4 = buds split and leaves visible, 5 = leaves open [on distal end of branches], 6 = leaves open over majority of the tree; adapted from Perfito et al. (2004)). We used these data to calculate median scores for each site.

The Abert’s towhee forages primarily on the ground (e.g., over 70% of foraging observations by Rosenberg et al. (1991) were on the ground). Furthermore, although this species consumes a variety of food types, arthropods dominate the diet in all seasons (Rosenberg et al., 1991). Therefore, to test whether food availability for Abert’s towhees differs between the two habitats, we quantified ground arthropod biomass using pitfall trap as in Cook and Faeth (2006). Pitfall traps consisted of two empty plastic cups (volume: 500 ml; height: 12 cm; diameter at opening: 9.5 cm), one inside the other and buried in the ground with the top of the cups slightly (∼5 mm) below the surface of the soil. The limitations of this method have been discussed elsewhere (Prasifka et al., 2007), but we assume that any bias in effectiveness is similar between the habitats. We placed 10 traps in each site (spaced 10 m apart), along the same 100 m transects as used for the tree phenology measurements. Traps were opened on the same day that tree phenology was scored and were left open for 72 h. After 72 h, we collected the content of each trap by emptying it into individual re-sealable plastic bags. Traps were then cleaned of all debris and blocked using caps between sampling periods. Bags containing trap contents were frozen later the same day at −20 °C until analysis. We sorted all arthropods, eliminated debris (e.g., sand and soil), and transferred the arthropods from a given trap into a plastic cup. Cup contents were dried in individual containers in an oven at 60 °C for 48 h and until mass was constant over 12 h. The entire arthropod content of each trap was then weighed to the nearest milligram, and an average dry arthropod biomass was calculated for each site at each sampling period.

### 2.4 Study 1 – The central control of reproduction and gonad development

The objective of study 1 was to test whether male urban and desert Abert’s towhees (*n* = 8 per habitat) differ in the amount of brain GnRH-1, GnIH, and NPY. Within 5 min of capture, each bird was deeply anesthetized by intramuscular (pectoral) injection of ketamine and xylazine (ketamine: 8 mg per 0.5 mL [160 mg/kg]; xylazine: 160 μg per 0.5 mL [3.2 mg/kg] dissolved in 0.9% NaCl solution). We then collected the brain and testes of each bird as previously described (Davies et al., 2015c). Briefly, birds were immediately transcardially perfused with wash solution (0.9% NaCl and 0.1% NaNO_2_ in 0.1 M phosphate buffer, PB), followed by 4% paraformaldehyde, and the brains and testes removed. Later the same day, we removed all extra connective tissue from the testes and then weighed them to the nearest 0.1 mg. Brains and testes were gelatin-embedded, cryoprotected, and stored at −80 °C until sectioned.

We coronally sectioned brains (30 μm thick sections) using a cryostat at −20 °C. Sections were divided into four parallel series by systematically alternating between four wells containing cryoprotectant solution. Sections were stored in cryoprotectant at −20 °C until immuno-labeled.

### 2.5 Immunohistochemistry

We immuno-labeled brain sections for GnRH-1, GnIH, and NPY immunoreactivity in three immunohistochemical runs per peptide. Each run included sections from 4 to 6 randomly selected birds. These protocols have previously been validated in our laboratory (NPY: Davies et al., 2015c; GnRH-I and GnIH: Small et al., 2008a), and the distribution of these peptides in the avian brain has been described in previous studies (summarized in Davies et al., 2015b). For details of the immunohistological procedure, see Davies et al. (2015c). For GnRH-1, we used anti-cGnRH-1 antiserum (6DL31/4 prepared by P.J. Sharp) at a dilution of 1:10,000. In the case of GnIH, we used anti-quail GnIH antiserum (Tsutsui et al., 2000) at a dilution of 1:5000. For NPY, we used anti-human/rat NPY antiserum (Bachem, Torrance, CA, USA) at a dilution of 1:10,000.

### 2.6 Immunohistochemistry data collection

All immunohistochemistry data were collected without knowledge of a bird’s capture location and by the same observer. For each bird, we counted the number of cells immunoreactive (ir) for GnRH-1, GnIH, and NPY present in each immune-labeled section. We analyzed sections across the whole region where each peptide is produced (i.e., preoptic area [POA], lateral hypothalamus [LHy], infundibular nucleus [IN], and paraventricular nucleus [PVN]; an average of 15 sections per bird for GnRH-1 and GnIH, and 9 sections for NPY). We then multiplied the number of immunoreactive cells by four to estimate the total number of cells for each bird. We quantified the area and optical density of GnRH-1 and GnIH immune-labeled perikarya as previously described (Davies et al., 2015c). Due to the dense network of NPY-ir fibers in the IN (Davies et al., 2015c), area and optical density could not be accurately quantified for these perikarya. Light intensity, aperture diameter, and camera shutter speed were standardized for all image captures. We photographed five perikarya from each section, which were randomly selected using a grid. Only perikarya for which the entire perimeter was unobstructed and clearly visible were used. Digitized images were analyzed using Image-Pro Plus (Media Cybernetics, LP, Silver Spring, Md., USA) by manually outlining each perikaryon and then determining the immuno-labeled area and optical density (arbitrary units: 0 = no staining, complete light transmission; and 1 = complete staining saturation, no light transmission). All images were standardized for individual variations in background immuno-labeling using Image-Pro’s background correction function.

To determine the density of GnRH-1-ir, GnIH-ir, and NPY-ir fibers in the median eminence (ME), we photographed two sections per brain. On the resulting image, we used Image-pro Plus to measure the optical density of five areas of interest (AOI, 65 × 65 μm each) per brain section. AOIs were evenly spaced from left to right along the ventral edge of the ME. We calculated an average optical density for each section, and then for each bird.

### 2.7 Testicular morphology

We sectioned the testes at a thickness of 30 μm using a cryostat at −21 °C and stored sections in 0.1 M PB until mounting on glass microscope slides later the same day. After allowing sections to dry at room temperature for 24 h, we stained them with hematoxylin and eosin (S212A and S176, Poly Scientific, Bay Shore, NY, USA) as previously described (Davies et al., 2015c).

Vernal reproductive development in many seasonally breeding birds involves a marked increase in testis size resulting from growth of seminiferous tubules. Thus, seminiferous tubule diameter is potentially a more sensitive indicator of testicular exocrine function than testis mass (Amann, 1986, Jenkins et al., 2007). We randomly selected eight sections from each bird (four from each testis) and, using Image Pro, measured the shortest diameter of 10 seminiferous tubules per section that were randomly selected using a grid overlaid on the image. These measurements were used to calculate an average seminiferous tubule diameter for each bird.

### 2.8 Habitat and inter-annual variation in gonad development and precipitation

To investigate the inter-annual variation in gonad growth between urban and desert birds, we compared CP width (a proxy for paired testis volume; see results) of birds in the current study (2013) with birds sampled in a previous study (2011 and 2012, Davies et al., 2015a) during the same period (i.e., March 14th to April 5th). We pooled data from 2011 and 2012 because CP width did not statistically differ between these years (Davies et al., 2015a). To better understand the ecological bases of inter-annual variation in gonad growth, we also compared precipitation at the study sites. Desert plant phenology is dependent on cumulative precipitation during the preceding 3 months (Buyantuyev and Wu, 2012). Therefore, we calculated the cumulative precipitation during January to March at three desert and five urban weather stations closest to our study sites. Precipitation data were obtained from the Flood Control District of Maricopa County, AZ (http://www.fcd.maricopa.gov/).

### 2.9 Study 2 – Endocrine responsiveness to GnRH challenge

The objective of study 2 was to determine whether sensitivity of the anterior pituitary gland and/or the gonads to a GnRH challenge differs between urban and desert Abert’s towhees. Within 2 min of collecting the initial blood sample described above, we randomly assigned birds (*n* = 8 per treatment per habitat; i.e., total sample size = 32) to receive an intravenous injection via the jugular vein of either synthetic chicken GnRH-1 (Sigma Chemical Co., MO, USA; at a dose equal to ≃25 μg/kg) in 0.1 ml saline solution (0.9% NaCl) or 0.1 ml saline (control). We collected two more blood samples (each ∼100 μl; as described above) from each bird 3 min and 20 min after the injection to determine post-injection plasma LH and T, respectively. Between the injection and the post-injection bleeds, birds were held in individual cloth bags. We selected the GnRH dose and the time between injection and post-injection blood samples based on their effectiveness to stimulate LH and T secretion in the Cassin’s sparrow, *Peucaea cassinii* (Deviche et al., 2012a). All birds received a uniquely numbered aluminum tarsal band from the U.S. Geological Survey and were released at the capture site.

### 2.10 Hormone assays

To quantify plasma LH, we used a micromodification of the radioimmunoassay described previously (Sharp et al., 1987). All samples were measured in a single assay and in duplicate. The intra-assay coefficient of variation was 3.6% and the minimum detectable dose was 0.2 ng/ml. This radioimmunoassay has been used to determine plasma LH in a range of bird species (Ciccone et al., 2007, Schaper et al., 2012a), including multiple emberizids (Deviche et al., 2012a, Deviche et al., 2012b, Meddle et al., 2002, Wingfield et al., 2012). We quantified plasma T concentration using enzyme-linked immunoassay (Enzo Life Sciences, Ann Arbor, MI, USA), which has been validated for Abert’s towhee in our laboratory by demonstrating parallelism of a serial plasma dilution (4× to 64× dilutions) with the standard curve using GraphPad Prism 5 (La Jolla, CA, USA). We assayed samples in duplicate and randomly assigned them to assay plates, except that all of the samples collected from any given individual were assayed on the same plate. Average assay sensitivity was 1.7 pg/ml. The average intra- and inter-assay coefficient of variation were 6.8% and 2.1%, respectively.

### 2.11 Statistical analysis

We compared the phenology of leaf foliage progression and arthropod dry biomass between the two habitats (i.e., urban vs. desert) using repeated measures ANOVA (rmANOVA) with sampling period as the within-subjects factor. In the case of leaf phenology, data were ranked before analysis and are presented as medians (±interquartile range; IQR). To analyze relationships between habitat and body mass, wing chord, body condition, furcular fat score, CP width, paired testis mass, seminiferous tubule diameter, and (log transformed) initial plasma LH and T we combined data from birds caught in study 1 and study 2, where applicable, and used Student’s *t*-tests or Mann–Whitney rank-sum tests when data were ordinal or not normally distributed. We tested whether CP widths of birds in the current study (2013) differed from those of birds sampled in 2011 and 2012 (Davies et al., 2015a) using a two-way ANOVA with habitat and year (i.e., 2011 and 2012 vs. 2013) as fixed factors. We analyzed cumulative precipitation using a rmANOVA. To analyze whether the number of brain cells immunoreactive for GnRH-1, GnIH, and NPY differed in urban and non-urban birds, we used Mann–Whitney rank-sum test. For the remaining measures of these neuropeptides (GnRH-1 and GnIH: cell body area and optical density, and optical density of fibers in the ME; NPY: density of fibers in the ME), we used Student’s *t*-tests (or Mann–Whitney rank-sum test when data were not normally distributed). To analyze the endocrine response (post-challenge concentration – initial concentration) to GnRH challenge, we used a two-way ANOVA with habitat and treatment (GnRH vs. saline) as fixed factors. We performed all statistical analyses using SPSS version 19 (Chicago, Illinois, USA) with *α* = 0.05. Post-hoc comparisons were performed using Tukey’s HSD test. Data are presented as mean (±SEM), unless otherwise stated.

## 3 Results

### 3.1 Plant and arthropod phenology

**Fig. 1:**
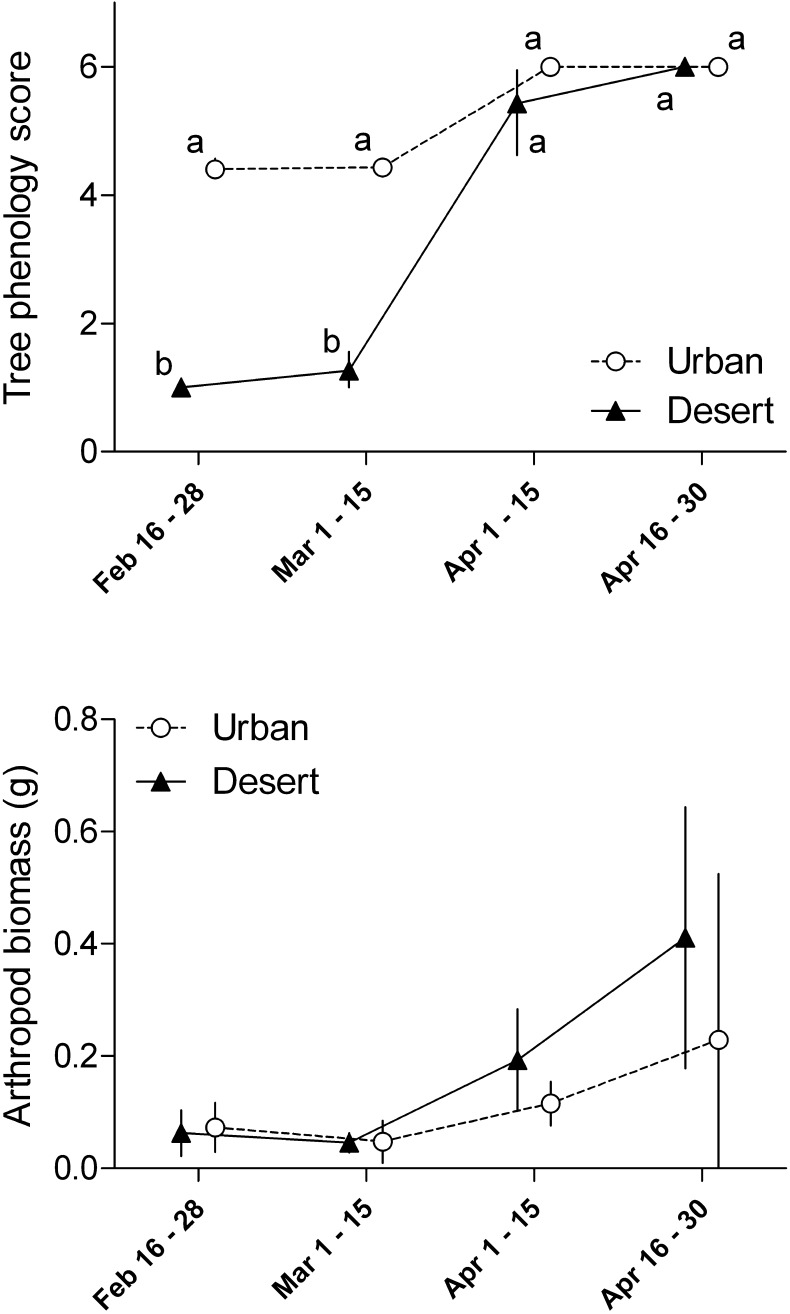
The phenology of tree leaf foliage progression, but not ground arthropod abundance, differed between urban and desert study sites. Tree leaf foliage progression of three species (mesquite, *Prosopis* spp.; palo verde, *Parkinsonia* spp.; and salt cedar, *Tamarix* spp.) common to both habitats was scored on a scale of 0–6. Ground arthropods were collected in empty pitfall traps and then dried before weighing to calculate dry biomass. The tree phenology panel depicts medians (±IQR), whereas the arthropod biomass panel depicts means (±SEM). Points with identical letters are not significantly different (*P* > 0.05). Both variables were measured along the same 100 m transects (4 urban sites; 3 desert sites) and collected at the same time, but datasets have been offset on the horizontal axis for visual clarity.

Plant phenology differed between the habitats (*F*_1,7_ = 57.84, *P* < 0.001) and there was an interaction of this factor with time (*F*_3,21_ = 6.74, *P* = 0.002; Fig. 1). Post hoc analysis showed that the median urban tree phenology score did not detectably change over the course of the study. The median tree phenology score of desert sites, on the other hand, was lower at the beginning of the study and increased over course of the spring to similar levels as urban trees. Ground arthropod dry biomass increased over the study period (*F*_3,21_ = 4.49, *P* = 0.014; Fig. 1). However, there was no significant difference between urban and desert sites (*F*_1,7_ = 0.077, *P* = 0.79), and no interaction between habitat and time (*F*_3,21_ = 0.99, *P* = 0.43; Fig. 1).

### 3.2 Morphometrics

Urban and desert birds from both studies did not significantly differ with respect to body mass (urban = 47.8 (±0.33) g; desert = 48.1 (±0.58) g; *t*_46_ = −0.50, *P* = 0.62) or wing chord (urban = 92.0 (±0.33) mm; desert = 91.3 (±0.54) mm; *t*_46_ = 1.15, *P* = 0.26). Body condition was likewise not significantly different between urban and desert birds (urban = 45.9 (±0.32) g; desert = 45.2 (±0.29) g; *t*_46_ = 1.51, *P* = 0.14). Furthermore, urban and desert towhees had similar furcular fat scores (median (±IQR): urban = 1 (±0); desert = 1 (±0); *U* = 288.0, *n*_1_ = 24, *n*_2_ = 24, *P* = 0.99) and CP widths (median (±IQR): urban: 7.28 (±0.62) mm; desert: 7.24 (±0.9) mm; *U* = 280.0, *n*_1_ = 24, *n*_2_ = 24, *P* = 0.88).

### 3.3 Study 1 – The central control of reproduction

#### 3.3.1 GnRH-1, GnIH, and NPY

Urban and desert birds did not significantly differ in the number of GnRH-1-ir cells in the POA (*U* = 40.0, *n*_1_ = 8, *n*_2_ = 8, *P* = 0.44) or the LHy (*U* = 35.5, *n*_1_ = 8, *n*_2_ = 8, *P* = 0.72; Table S1). Likewise, the total number of GnRH-1-ir cells (i.e., POA plus LHy) did not significantly differ between birds from the two habitats (*U* = 39.0, *n*_1_ = 8, *n*_2_ = 8, *P* = 0.50; Table S1). There was no significant difference between urban and desert towhees in POA GnRH-1-ir cell body area (*t*_14_ = −1.91, *P* = 0.077) and optical density (*t*_14_ = −0.33, *P* = 0.75), and optical density of GnRH-1-ir fibers in the ME (*t*_14_ = −1.07, *P* = 0.30; Table S1).

Urban and desert birds did not significantly differ in the number of GnIH-ir cells in the PVN (*U* = 36.0, *n*_1_ = 8, *n*_2_ = 8, *P* = 0.72; Table S1). Furthermore, urban and desert birds did not significantly differ in GnIH-ir cell body area (*t*_14_ = −0.32, *P* = 0.76) and optical density (*U* = 38.0, *n*_1_ = 8, *n*_2_ = 8, *P* = 0.57), and optical density of GnIH-ir fibers in the ME (*t*_14_ = 0.47, *P* = 0.65; Table S1). Likewise, the number of NPY-ir cells in the IN (*U* = 60.0, *n*_1_ = 8, *n*_2_ = 8, *P* = 0.44) and the optical density of NPY-ir fibers in the ME (*U* = 32.0, *n*_1_ = 8, *n*_2_ = 8, *P* = 0.96; Table S1) did not detectably differ between urban and desert towhees.

### 3.4 Initial endocrine activity and gonad growth

**Fig. 2:**
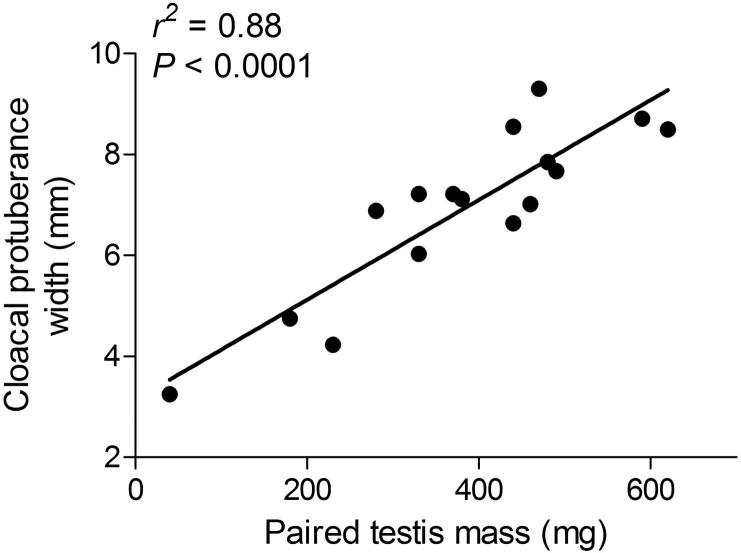
Cloacal protuberance width of free-ranging adult male Abert’s towhees, *Melozone aberti*, is positively correlated with paired testis mass (*r*^2^ = 0.88, *P* < 0.0001). Each point represents one individual.

**Fig. 3:**
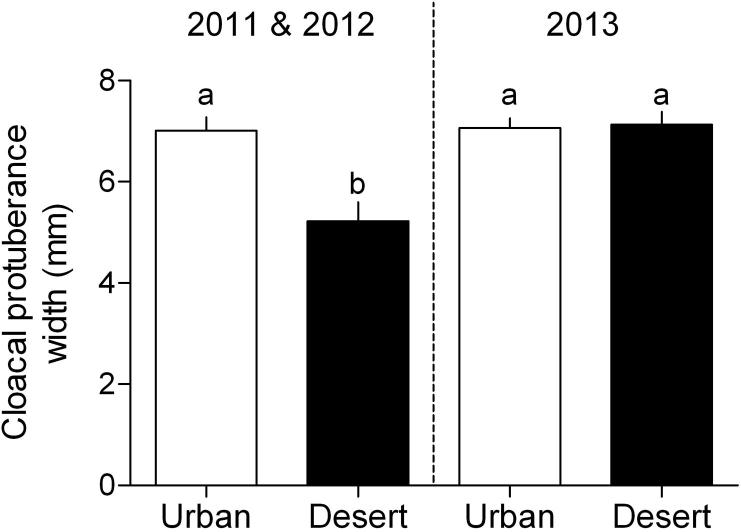
Desert, but not urban, adult male Abert’s towhees, *Melozone aberti*, advanced the timing of seasonal reproductive development in 2013 compared to 2011 and 2012. The cloacal protuberance width of urban towhees in the current study (2013) was similar to that of birds from the same population sampled in a previous study (Davies et al., 2015a). By contrast, cloacal protuberance width of desert towhees was larger in the current study compared to that of birds from the same population sampled in Davies et al. (2015a) Data points are means ± SEM, and points with identical letters are not significantly different (*P* > 0.05; Tukey HSD test).

**Fig. 4:**
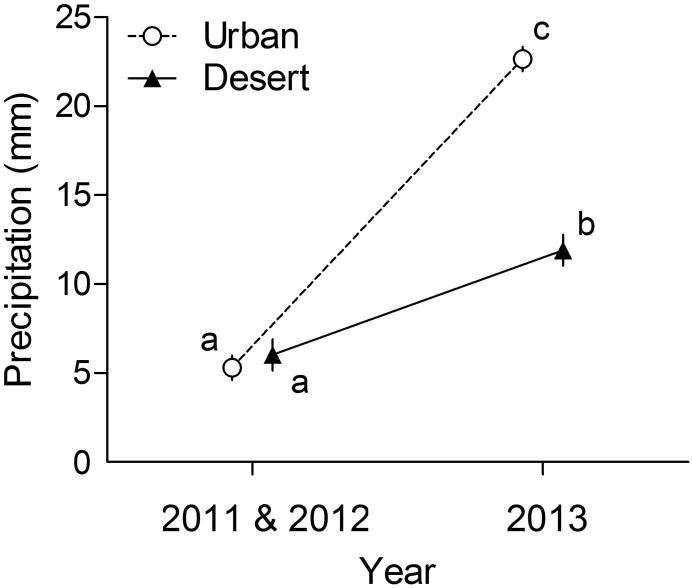
In the current study (2013), precipitation was higher compared to the previous two years (2011 and 2012, Davies et al., 2015a) and was higher in urban compared to desert locations. We compared the cumulative precipitation of three desert and five urban weather stations during January to March. Points are means and error bars show SEM, and points with identical letters are not statistically different. For visual clarity, points have been offset on the horizontal axis.

Initial plasma LH and T of towhees in study 1 also did not significantly differ between the two habitats (LH: *t*_14_ = −1.52, *P* = 0.15; T: *t*_14_ = −0.07, *P* = 0.94; Table S2). Urban and desert birds had similar testis masses (*t*_14_ = 0.88, *P* = 0.39) and seminiferous tubule diameters (*t*_14_ = 1.94, *P* = 0.07; Table S2). Testis mass was positively related to CP width (*r*^2^ = 0.88, *P* < 0.0001; Fig. 2). Cloacal protuberance width differed between birds in the current study versus those of Davies et al. (2015a) (*F*_1,71_ = 11.63, *P* = 0.001). Furthermore, there was a difference between birds from the two habitats (*F*_1,71_ = 6.60, *P* = 0.012), and a two-way interaction between the study year and habitat (*F*_1,71_ = 10.34, *P* = 0.002; Fig. 4). Cloacal protuberance width of urban towhees did not significantly differ between the two studies (Tukey HSD: *P* > 0.05; Fig. 3). By contrast, desert birds had larger CP in the current than the previous study (Tukey HSD: *P* < 0.001; Fig. 3).

Cumulative precipitation during January to March differed between the two habitats (*F*_1,15_ = 15.42, *P* = 0.008) and between years (*F*_1,15_ = 218.17, *P* < 0.001), and there was an interaction between these factors (*F*_1,15_ = 53.16, *P* < 0.001; Fig. 4). Precipitation did not significantly differ between urban and desert locations in 2011 and 2012 (*P*’s = 0.64; Fig. 4). However, precipitation was higher during 2013 than 2011 and 2012, and also higher in the urban than desert locations (Tukey HSD: *P* < 0.001; Fig. 4).

### 3.5 Study 2 – Endocrine responsiveness to GnRH challenge

Initial plasma LH and T of birds in study 2 did not significantly differ between the habitats (LH: *F*_1,28_ = 0.49, *P* = 0.49; T: *F*_1,28_ = 0.74, *P* = 0.40) and in birds from the two treatment groups (LH: *F*_1,28_ = 1.07, *P* = 0.31; T: *F*_1,28_ = 0.18, *P* = 0.67; Table S3). Furthermore, there was no interaction between habitat and treatment group on initial plasma LH (*F*_1,28_ = 2.08, *P* = 0.16) or T (*F*_1,28_ = 0.59, *P* = 0.45). As expected, GnRH challenge elicited an increase in both plasma LH and T (Tukey HSD: *P*’s < 0.001), whereas saline was associated with no change in plasma LH (Tukey HSD: *P* = 0.49) and a decrease in plasma T (Tukey HSD: *P* < 0.001; treatment × time interaction: LH: *F*_1,28_ = 26.73, *P* < 0.0001; T: *F*_1,28_ = 63.11, *P* < 0.0001).

**Fig. 5:**
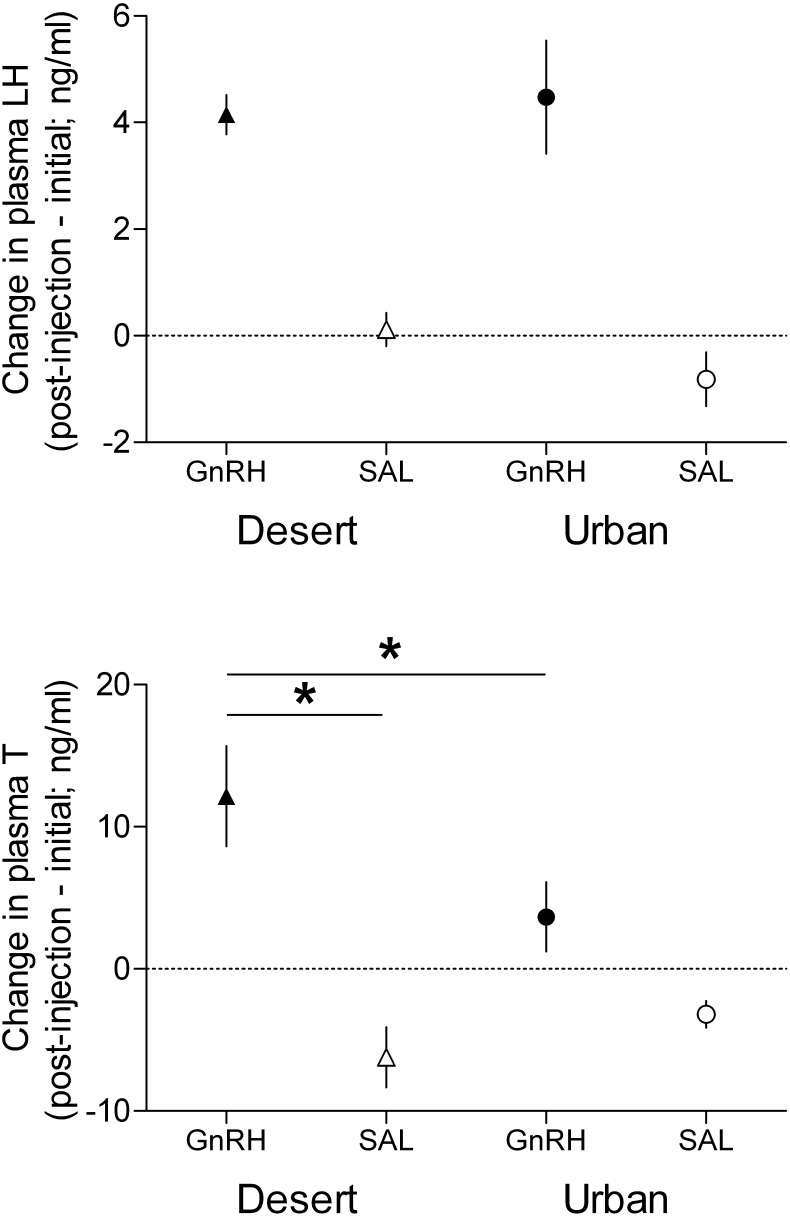
The plasma luteinizing hormone (LH) response to GnRH challenge did not significantly differ between urban and desert Abert’s towhees, *Melozone aberti*, but the plasma testosterone (T) response was greater in desert compared to urban towhees (*n* = 8 per group). Free-ranging adult males were bled within 3 min of capture, received an intravenous injection of gonadotropin-releasing hormone (GnRH) or vehicle (saline; SAL), and were bled again 3 min (LH) and 20 min (T) later (post-injection). Points are means (post-challenge – initial) and error bars show SEM. Asterisks denote significant differences between groups (Tukey HSD: *P* < 0.05).

There was no overall effect of habitat on the response of either plasma LH or T to treatment (LH: *F*_1,28_ = 0.23, *P* = 0.64; T: *F*_1,28_ = 1.27, *P* = 0.27). There was no interaction between treatment and habitat on the change in plasma LH (*F*_1,28_ = 0.98, *P* = 0.33; Fig. 5), but the change in plasma T was significantly affected by the two-way interaction (*F*_1,28_ = 5.59, *P* = 0.025; Fig. 5). This difference resulted from a greater plasma T response to GnRH challenge of desert towhees compared to that of urban towhees (Tukey HSD: *P* = 0.021; Fig. 5). Desert towhees differed in their plasma T response to GnRH challenge compared to saline injection (Tukey HSD: *P* < 0.001). By contrast, in urban towhees there was only a (non-significant) trend toward a difference between the plasma T responses to GnRH challenge and to saline injection (Tukey HSD: *P* = 0.056).

## 4 Discussion

The available evidence suggests that birds adjust to urban areas by advancing the phenology of vernal gonad growth (Davies et al., 2015a, Deviche et al., 2010, Deviche and Davies, 2014, Partecke et al., 2005). However, the underlying cause of fine-scale variation in the phenology of gonad growth remains unclear. Our previous study on Abert’s towhees (from the same locations as this study) found that urban males advanced the phenology of vernal gonad growth and plasma LH secretion, relative to desert males (Davies et al., 2015a), so our finding that these populations did not differ in any measure of baseline reproductive (neuro)endocrine activity during the 2013 breeding period was unexpected. Specifically, we found no difference in any measure of the central control of reproduction (i.e., hypothalamic GnRH-1 and GnIH), in any measure of gonad growth or baseline endocrine activity (i.e., paired testis mass, seminiferous tubule diameter, baseline plasma LH and T), or in a secondary sexual characteristic (CP width). Interestingly, the inter-annual comparison of CP width, which is positively related to paired testis mass, indicates that, in the three years considered here, there is limited inter-annual variation in the phenology of gonad growth in urban Abert’s towhees. By contrast, in nearby populations of desert Abert’s towhees, there is considerable inter-annual variation, whereby desert towhees advanced the phenology of gonad growth in the current year relative to previous years. This finding has implications for our understanding of both the ecological and physiological causes of variation in the phenology of gonad growth in urban and non-urban birds.

### 4.1 Ecological control of gonad growth phenology

We hypothesized that greater food abundance in urban areas of Phoenix contributes to the advanced phenology of gonad growth seen in urban towhees (Deviche and Davies, 2014). However, in contradiction to this hypothesis, we found that ground arthropod biomass did not differ between urban and desert locations of Phoenix, suggesting that food abundance is the same for towhees regardless of location. Consistent with this result, measures of energetic status (body condition and fat stores) and hypothalamic levels of NPY, a neuropeptide that, in mammalian models, links food availability to endocrine activity of the HPG axis, were similar in urban and desert towhees. Given that the phenology of gonad growth (as indicated by CP width) was advanced only in the previous study (Davies et al., 2015a), but energetic status did not differ between the two populations in either study, there is little evidence to support the hypothesis that differences in energetic status due to greater food abundance in urban areas of Phoenix drives the disparity in gonad growth phenology between urban and desert Abert’s towhees.

An alternative hypothesis to explain the observed inter-annual variation in gonad growth phenology between urban and desert towhees is that the inter-annual variation in the phenology of key food types differs between urban and desert locations of Phoenix (Davies et al., 2015a, Deviche and Davies, 2014). Under this hypothesis, the overall abundance of food is similar between urban and desert areas, but the phenology of growing green vegetation and arthropods, which birds may use as a supplementary cue to optimally time gonad growth, may be more consistent from year-to-year in urban areas, relative to desert areas. The presence and/or consumption of preferred food types accelerates gonad growth in a variety of songbirds (Ettinger and King, 1981, Hahn et al., 2005, Hau et al., 2000, Voigt et al., 2007, Watts and Hahn, 2012). Furthermore, the ecology of the Sonoran Desert suggests that there may be considerable variation in the phenology of seasonally available food types both inter-annually and between urban and desert areas. In arid regions like the Sonoran Desert, the phenology of vernal plant growth is highly dependent on winter precipitation (Bowers and Dimmitt, 1994, Noy-Meir, 1973, Schwinning et al., 2004), the amounts of which vary unpredictably from year to year. In years with high winter precipitation, plant growth is advanced, whereas in years with low precipitation, plant growth is delayed (Bowers and Dimmitt, 1994, Noy-Meir, 1973). By contrast, due to anthropogenic resource input (in this case, principally irrigation water), plant growth in urban areas of Phoenix is decoupled from precipitation (Buyantuyev and Wu, 2012). Consequently, urbanization of Phoenix is associated with reduced inter- and intra-annual variability in plant growth phenology, relative to desert areas. This is in accordance with our finding that the growing season of three tree species in urban areas had already begun (as indicated by the presence of green leaves) at the start our study and exhibited no detectable change. These tree species in desert areas, on the other hand, were inactive at the start of our study and demonstrated a marked seasonal increase in leaf phenology. Reduced inter-annual variability in urban plant growth phenology is also in accordance with our inter-annual comparison of gonad growth phenology in Abert’s towhees. Like plant growing seasons, urban towhees also appear to have limited inter-annual variability in the timing of gonad growth, despite considerable variability in winter precipitation. By contrast, the inter-annual variability of gonad growth phenology in desert towhees corresponds with the variation in winter precipitation. In our previous study (Davies et al., 2015a), winter precipitation was low and the phenology of gonad growth in desert birds was delayed, whereas in the current study precipitation was higher and the phenology of gonad growth was advanced. We suggest that the phenology of trees in desert areas was relatively early in the current study, which may account for lack of habitat-related difference in gonad growth phenology of Abert’s towhees.

Many songbird populations synchronize the breeding period with the peak in arthropod, especially caterpillar, availability (Cresswell and Mccleery, 2003, Visser et al., 2006), which, in turn, is synchronized with tree phenology (van Asch and Visser, 2007, Visser et al., 2006). We, therefore, predict that the phenology of arthropod availability in urban areas of Phoenix parallels the phenology of urban plants and has limited inter-annual variability. The phenology of arthropods in desert areas, on the other hand, exhibits greater inter-annual variability. If birds use the timing of growing green vegetation and/or arthropods as environmental cues to optimally time gonad development, this may account for the disparity in inter-annual variability seen between urban and desert Abert’s towhees. Specifically, in years with high winter precipitation, the phenology of plants and arthropods in desert areas will be relatively early and Abert’s towhees in these areas will begin gonad development relatively early. In years with low winter precipitation, the phenology of plants, arthropods, and, in turn, gonad growth will be delayed. In contrast, the phenology of urban plants, arthropods, and Abert’s towhee gonad growth is independent of precipitation patterns and is relatively constant from year-to-year. We suggest that future comparative studies of gonad growth phenology of urban and non-urban birds may find it illuminating to also compare the phenology of plants and potential food sources, and to study these factors over multiple years with variable environmental conditions. An additional, but not mutually exclusive, hypothesis to account for the inter-annual variation in gonad growth phenology between urban and desert towhees is that artificial light in urban areas advances the initiation of gonad development (Kempenaers et al., 2010). Our finding that the timing of gonadal maturation of urban birds is relatively constant from year-to-year cannot exclude a role for artificial light advancing gonadal development within the window of reproductive development set by other environmental cues. It remains unclear, however, whether artificial light directly stimulates the HPG axis via hypothalamic encephalic receptors and leads to a perception of unnaturally longer day lengths (Deviche and Davies, 2014).

### 4.2 Physiological control of gonad growth phenology

Our hypothesized disparity in the seasonality of supplementary environmental cues between urban and desert areas of Phoenix may also account for the observed habitat-related difference in responsiveness of the gonads to GnRH challenge. Desert towhees inhabit an environment with an unpredictable and marked seasonal change in growing green vegetation (and possibly also arthropod availability), whereas urban birds inhabit an environment with a more predictable and limited change in these factors (Bowers and Dimmitt, 1994, Buyantuyev and Wu, 2012, Noy-Meir, 1973). We found that the plasma LH response to GnRH challenge was similar between the two habitats, whereas this treatment elicited a marked increase in plasma T in desert, but not urban, towhees. This raises the intriguing possibility that, in response to the habitat-related disparity in the predictability and magnitude of change in plant and arthropod phenology, Abert’s towhees have adjusted the responsiveness of the gonads to environmental stimuli that would naturally elicit an increase in GnRH-1 and, in turn, LH secretion. A range of supplementary environmental cues, such as the availability of preferred food types (Hahn et al., 2005, Hau et al., 2000), precipitation (Small et al., 2008a), and song (Small et al., 2008b, Wingfield and Wada, 1989), can rapidly elicit an increase in endocrine activity of the HPG axis. It may be beneficial for desert towhees to rapidly respond to the unpredictable and marked seasonal increase in plant and arthropod phenology and initiate gonad growth. By contrast, it may be less important for urban birds to respond as rapidly due to the more predictable and less marked seasonal increase in urban plant and arthropod phenology. An alternative, not mutually exclusive explanation for the habitat-related difference in the response to GnRH challenge is that gonadal responsiveness is positively related to the intensity of territorial aggression. Specifically, compared to urban towhees, desert towhees may have a larger plasma T response to endocrine stimulation, possibly due to higher LH receptor density in the gonads, in order to elicit more intense aggressive responses to future territorial intrusions. If this hypothesis is correct, however, then further studies are necessary to address the counterintuitive finding that urban towhees are more aggressive than desert towhees (Fokidis et al., 2011), but have a weaker response to GnRH challenge. Consistent with previous studies on other birds (Deviche et al., 2012b), our finding that plasma T decreased 20 min after saline injection suggests that the acute stress of capture and restraint elicits a rapid decline in plasma levels of this hormone in male Abert’s towhees. Future studies are warranted so that comparisons of the response to GnRH challenge of urban and non-urban birds can be made at multiple times during vernal gonad growth while quantifying the phenology of plants and arthropods.

### 4.3 Conclusions

The findings of the current study demonstrate that gonad growth phenology is not always advanced in urban birds, relative to their desert conspecifics. In three years that differed in the habitat-related disparity in gonad growth, energetic status did not significantly differ between the two populations at any time. This finding provides no support for the hypothesis that greater food abundance in urban areas of Phoenix drives the habitat-related disparity in gonad growth phenology. Our results are instead consistent with the hypothesis that differences in the predictability and magnitude of change in supplementary environmental cues, particularly the availability of key food sources, between urban and desert areas of Phoenix contributes to the observed habitat-related disparity in inter-annual variability in gonad growth. The physiological mechanism responsible for this phenomenon appears to involve the initial endocrine activity of the anterior pituitary gland and potentially also the endocrine responsiveness of the gonads. Our findings highlight the need for future investigations into the adjustment of gonad growth and initial endocrine activity by birds to urban areas to include multiple years with a range of environmental conditions.

## Appendix A Supplementary data

Supplementary data 1 This document file contains Supplementary “Tables S1–S3”.

## References

[b0005] Amann R.P. (1986). Detection of alterations in testicular and epididymal function in laboratory animals. Environ. Health Perspect..

[b0010] Boswell T., Dawson A., Chaturvedi C.M. (2001). Regulation of feeding by neuropeptide Y. Avian Endocrinology.

[b0015] Bowers J.E., Dimmitt M.A. (1994). Flowering phenology of six woody plants in the northern Sonoran Desert. Bull. Torrey Bot. Club.

[b0020] Bubenik G.A., Schams D., White R.J., Rowell J., Blake J., Bartos L. (1997). Seasonal levels of reproductive hormones and their relationship to the antler cycle of male and female reindeer (*Rangifer tarandus*). Comp. Biochem. Physiol. B.

[b0025] Buyantuyev A., Wu J. (2010). Urban heat islands and landscape heterogeneity: linking spatiotemporal variations in surface temperatures to land-cover and socioeconomic patterns. Landscape Ecol..

[b0030] Buyantuyev A., Wu J. (2012). Urbanization diversifies land surface phenology in arid environments: interactions among vegetation, climatic variation, and land use pattern in the Phoenix metropolitan region, USA. Landscape Urban Planning.

[b0035] Caro S.P., Lambrechts M.M., Chastel O., Sharp P.J., Thomas D.W., Balthazart J. (2006). Simultaneous pituitary–gonadal recrudescence in two Corsican populations of male blue tits with asynchronous breeding dates. Horm. Behav..

[b0040] Caro S.P., Schaper S.V., Hut R.A., Ball G.F., Visser M.E. (2013). The case of the missing mechanism: how does temperature influence seasonal timing in endotherms?. PLoS Biol..

[b0045] Ciccone N.A., Dunn I.C., Sharp P.J. (2007). Increased food intake stimulates GnRH-I, glycoprotein hormone [alpha]-subunit and follistatin mRNAs, and ovarian follicular numbers in laying broiler breeder hens. Domest. Anim. Endocrinol..

[b0050] Clarke I.J., Parkington H.C. (2013). Gonadotropin inhibitory hormone (GnIH) as a regulator of gonadotropes. Mol. Cell. Endocrinol..

[b0055] Cook W.M., Faeth S.H. (2006). Irrigation and land use drive ground arthropod community patterns in an urban desert. Environ. Entomol..

[b0060] Cresswell W., Mccleery R. (2003). How great tits maintain synchronization of their hatch date with food supply in response to long-term variability in temperature. J. Anim. Ecol..

[b0065] Davies S., Rodriguez N.S., Sweazea K.L., Deviche P. (2013). The effect of acute stress and long-term corticosteroid administration on plasma metabolites in an urban and desert songbird. Physiol. Biochem. Zool..

[b0070] Davies S., Deviche P. (2014). At the crossroads of physiology and ecology: food supply and the timing of avian reproduction. Horm. Behav..

[b0075] Davies S., Behbahaninia H., Giraudeau M., Meddle S.L., Waites K., Deviche P. (2015). Advanced seasonal reproductive development in a male urban bird is reflected in earlier plasma luteinizing hormone rise but not energetic status. Gen. Comp. Endocrinol..

[b0080] Davies S., Gao S., Valle S., Bittner S., Hutton P., Meddle S.L., Deviche P. (2015). Negative energy balance in a male songbird, the Abert’s towhee, constrains the testicular endocrine response to luteinizing hormone stimulation. J. Exp. Biol..

[b0085] Davies S., Cros T., Richard D., Meddle S.L., Tsutsui K., Deviche P. (2015). Food availability, energetic constraints and reproductive development in a wild seasonally breeding songbird. Funct. Ecol..

[b0090] Dawson A. (2008). Control of the annual cycle in birds: endocrine constraints and plasticity in response to ecological variability. Philos. Trans. R. Soc. B.

[b0095] Dawson A. (1983). Plasma gonadal steroid levels in wild starlings (*Sturnus vulgaris*) during the annual cycle and in relation to the stages of breeding. Gen. Comp. Endocrinol..

[b0100] Deviche P., Hurley L.L., Fokidis H.B., Norris D.O., Lopez K.H. (2010). Avian testicular structure, function, and regulation. Hormones and Reproduction of Vertebrates.

[b0105] Deviche P., Dawson A., Sabo J., Fokidis B., Davies S., Hurley L. (2012). Up to the challenge? Hormonal and behavioral responses of free-ranging male Cassin’s sparrows, *Peucaea cassinii*, to conspecific song playback. Horm. Behav..

[b0110] Deviche P., Gao S., Davies S., Sharp P.J., Dawson A. (2012). Rapid stress-induced inhibition of plasma testosterone in free-ranging male rufous-winged sparrows, *Peucaea carpalis*: characterization, time course, and recovery. Gen. Comp. Endocrinol..

[b0115] Deviche P., Davies S., Gil D., Brumm H. (2014). Reproductive phenology of urban birds: environmental cues and mechanisms. Avian Urban Ecology: Behavioral and Physiological Adaptations.

[b0120] Dornas R.A.P., Oliveira A.G., Dias M.O., Mahecha G.A.B., Oliveira C.A. (2008). Comparative expression of androgen receptor in the testis and epididymal region of roosters (*Gallus domesticus*) and drakes (*Anas platyrhynchos*). Gen. Comp. Endocrinol..

[b0125] Ettinger A.O., King J.R. (1981). Consumption of green wheat enhances photostimulated ovarian growth in white-crowned sparrows. Auk.

[b0130] Fokidis H.B., Orchinik M., Deviche P. (2011). Context-specific territorial behavior in urban birds: no evidence for involvement of testosterone or corticosterone. Horm. Behav..

[b0135] Hahn T.P., Pereyra M.E., Katti M., Ward G.M., MacDougall-Shackleton S.A., Dawson A., Sharp P.J. (2005). Effects of food availability on the reproductive system. Functional Avian Endocrinology.

[b0140] Hau M., Wikelski M., Wingfield J.C. (2000). Visual and nutritional food cues fine-tune timing of reproduction in a neotropical rainforest bird. J. Exp. Zool..

[b0145] Itoh M., Inoue M., Ishii S. (1990). Annual cycle of pituitary and plasma gonadotropins and plasma sex steroids in a wild population of the toad, *Bufo japonicus*. Gen. Comp. Endocrinol..

[b0150] Jawor J.M., McGlothlin J.W., Casto J.M., Greives T.J., Snajdr E.A., Bentley G.E., Ketterson E.D. (2006). Seasonal and individual variation in response to GnRH challenge in male dark-eyed juncos (*Junco hyemalis*). Gen. Comp. Endocrinol..

[b0155] Jawor J.M., McGlothlin J.W., Casto J.M., Greives T.J., Snajdr E.A., Bentley G.E., Ketterson E.D. (2007). Testosterone response to GnRH in a female songbird varies with stage of reproduction: implications for adult behaviour and maternal effects. Funct. Ecol..

[b0160] Jenkins L.K., Ross W.L., Young K.A. (2007). Increases in apoptosis and declines in Bcl-XL protein characterise testicular regression in American crows (*Corvus brachyrhynchos*). Reprod. Fertil. Dev..

[b0165] Kempenaers B., Borgström P., Loës P., Schlicht E., Valcu M. (2010). Artificial night lighting affects dawn song, extra-pair siring success, and lay date in songbirds. Curr. Biol..

[b0170] Kuenzel W.J. (2000). Central nervous system regulation of gonadal development in the avian male. Poult. Sci..

[b0175] Lourdais O., Bonnet X., Shine R., DeNardo D., Naulleau G., Guillon M. (2002). Capital-breeding and reproductive effort in a variable environment: a longitudinal study of a viviparous snake. J. Anim. Ecol..

[b0180] Maney D.L., Goode C.T., Ball G.F. (2007). Transduction of a non-photic cue: from the auditory system to a neuroendocrine response?. J. Ornithol..

[b0185] Marty N., Dallaporta M., Thorens B. (2007). Brain glucose sensing, counterregulation, and energy homeostasis. Physiology.

[b0190] McGlothlin J.W., Whittaker D.J., Schrock S.E., Gerlach N.M., Jawor J.M., Snajdr E.A., Ketterson E.D., Ketterson E.D. (2010). Natural selection on testosterone production in a wild songbird population. Am. Nat..

[b0195] Meddle S.L., Romero L.M., Astheimer L.B., Buttemer W.A., Moore I.T., Wingfield J.C. (2002). Steroid hormone interrelationships with territorial aggression in an arctic-breeding songbird, Gambel’s white-crowned sparrow, *Zonotrichia leucophrys gambelii*. Horm. Behav..

[b0200] Miner B.G., Sultan S.E., Morgan S.G., Padilla D.K., Relyea R.A. (2005). Ecological consequences of phenotypic plasticity. Trends Ecol. Evol..

[b0205] Noy-Meir I. (1973). Desert ecosystems: environment and producers. Annu. Rev. Ecol. Syst..

[b0210] Palmer S.S., Nelson R.A., Ramsay M.A., Stirling I., Bahr J.M. (1988). Annual changes in serum sex steroids in male and female black (*Ursus americanus*) and polar (*Ursus maritimus*) bears. Biol. Reprod..

[b0215] Partecke J., Van’t Hof T., Gwinner E. (2005). Underlying physiological control of reproduction in urban and forest-dwelling European blackbirds *Turdus merula*. J. Avian Biol..

[b0220] Peig J., Green A.J. (2009). New perspectives for estimating body condition from mass/length data: the scaled mass index as an alternative method. Oikos.

[b0225] Penfold L., Wildt D.E., Herzog T., Lynch W., Ware L., Derrickson S., Monfort S.L. (2001). Seasonal patterns of LH, testosterone and semen quality in the Northern pintail duck (*Anas acuta*). Reprod. Fertil. Dev..

[b0230] Perfito N., Tramontin A.D., Meddle S., Sharp P., Afik D., Gee J., Ishii S., Kikuchi M., Wingfield J.C. (2004). Reproductive development according to elevation in a seasonally breeding male songbird. Oecologia.

[b0235] Perfito N., Kwong J.M.Y., Bentley G.E., Hau M. (2008). Cue hierarchies and testicular development: is food a more potent stimulus than day length in an opportunistic breeder (*Taeniopygia g. guttata*)?. Horm. Behav..

[b0240] Prasifka J.R., Lopez M.D., Hellmich R.L., Lewis L.C., Dively G.P. (2007). Comparison of pitfall traps and litter bags for sampling ground-dwelling arthropods. J. Appl. Entomol..

[b0245] Rosenberg K.V., Ohmart R.D., Hunter W.C., Anderson B.W. (1991). Birds of the Lower Colorado River Valley.

[b0250] Schaper S.V., Dawson A., Sharp P.J., Caro S.P., Visser M.E. (2012). Individual variation in avian reproductive physiology does not reliably predict variation in laying date. Gen. Comp. Endocrinol..

[b0255] Schaper S.V., Dawson A., Sharp P.J., Gienapp P., Caro S.P., Visser M.E. (2012). Increasing temperature, not mean temperature, is a cue for avian timing of reproduction. Am. Nat..

[b0260] Schwinning S., Sala O.E., Loik M.E., Ehleringer J.R. (2004). Thresholds, memory, and seasonality: understanding pulse dynamics in arid/semi-arid ecosystems. Oecologia.

[b0265] Sharp P.J. (2005). Photoperiodic regulation of seasonal breeding in birds. Ann. N. Y. Acad. Sci..

[b0270] Sharp P.J., Dunn I.C., Talbot R.T. (1987). Sex differences in the LH responses to chicken LHRH-I and -II in the domestic fowl. J. Endocrinol..

[b0275] Small T.W., Sharp P.J., Bentley G.E., Millar R.P., Tsutsui K., Mura E., Deviche P. (2008). Photoperiod-independent hypothalamic regulation of luteinizing hormone secretion in a free-living Sonoran Desert bird, the Rufous-Winged Sparrow (*Aimophila carpalis*). Brain Behav. Evol..

[b0280] Small T.W., Sharp P.J., Bentley G.E., Millar R.P., Tsutsui K., Strand C., Deviche P. (2008). Auditory stimulation of reproductive function in male rufous-winged sparrows, *Aimophila carpalis*. Horm. Behav..

[b0285] Tsutsui K., Saigoh E., Ukena K., Teranishi H., Fujisawa Y., Kikuchi M., Ishii S., Sharp P.J. (2000). A novel avian hypothalamic peptide inhibiting gonadotropin release. Biochem. Biophys. Res. Commun..

[b0290] Tsutsui K., Ubuka T., Bentley G.E., Kriegsfeld L.J. (2013). Review: regulatory mechanisms of gonadotropin-inhibitory hormone (GnIH) synthesis and release in photoperiodic animals. Front. Neurosci..

[b0295] Tweit R.C., Finch D.M., Poole A. (1994). Abert’s towhee (*Melozone aberti*). The Birds of North America.

[b0300] van Asch M., Visser M.E. (2007). Phenology of forest caterpillars and their host trees: the importance of synchrony. Annu. Rev. Entomol..

[b0305] Visser M.E., Holleman L.J.M., Gienapp P. (2006). Shifts in caterpillar biomass phenology due to climate change and its impact on the breeding biology of an insectivorous bird. Oecologia.

[b0310] Voigt C., Goymann W., Leitner S. (2007). Green matters! Growing vegetation stimulates breeding under short-day conditions in wild canaries (*Serinus canaria*). J. Biol. Rhythms.

[b0315] Watts H.E., Hahn T.P. (2012). Non-photoperiodic regulation of reproductive physiology in the flexibly breeding pine siskin (*Spinus pinus*). Gen. Comp. Endocrinol..

[b0320] Williams T.D. (2008). Individual variation in endocrine systems: moving beyond the ‘tyranny of the Golden Mean’. Philos. Trans. R. Soc. Lond. B Biol. Sci..

[b0325] Wingfield J.C., Kenagy G.J., Pang P.K.T., Schreibman M.P. (1986). Natural regulation of reproductive cycles. Vertebrate Endocrinology: Fundamentals and Biomedical Implications.

[b0330] Wingfield J.C., Sullivan K., Jaxion-Harm J., Meddle S.L. (2012). The presence of water influences reproductive function in the song sparrow (*Melospiza melodia morphna*). Gen. Comp. Endocrinol..

[b0335] Wingfield J.C., Wada M. (1989). Changes in plasma levels of testosterone during male-male interactions in the song sparrow, *Melospiza melodia*: time course and specificity of response. J. Comp. Physiol. A..

[b0340] Wingfield J.C. (2008). Organization of vertebrate annual cycles: implications for control mechanisms. Philos. Trans. R. Soc. B.

