# Supplementary material for: The ecological and physiological bases of variation in the phenology of gonad growth in an urban and desert songbird
Source: Gen Comp Endocrinol. 2016 May 1;230-231:17–25. doi: 10.1016/j.ygcen.2016.03.013 (PMC4890648; doi:10.1016/j.ygcen.2016.03.013)
Supplement: Supplementary data 1 — This document file contains Supplementary “Tables S1–S3”. [file mmc1.docx]

**Supplementary material for ‘The ecological and physiological bases of variation in the phenology of gonad growth in an urban and desert songbird’**

# Scott Davies ^a,b^*, Samuel Lane ^a^, Simone L. Meddle ^c^, Kazuyoshi Tsutsui ^d^, and Pierre Deviche ^a^

^a^ *School of Life Sciences,* *Arizona State University, Tempe, Arizona 85287, USA*

^b^ *Present address: Department of Biological Sciences, Virginia Tech, Blacksburg, Virginia*

*10 24061, USA*

^c^ *The Roslin Institute & Royal (Dick) School of Veterinary Studies, University of Edinburgh, Roslin, Midlothian EH25 9PS, UK*

^d^ *Laboratory of Integrative Brain Sciences, Department of Biology and Center for Medical Life Science, Waseda University, Tokyo 162-8480, Japan*

**Table S1.** The number, area, and optical density of cell bodies, and optical density of fibers in the median eminence (ME), immunolabeled for three neuropeptides (gonadotropin-releasing hormone-1 (GnRH), gonadotropin-inhibitory hormone (GnIH), and neuropeptide Y (NPY)) of free-ranging urban (*n* = 8) and desert (*n* = 8) adult male Abert’s towhees, *Melozone aberti*. The total number of cells immunoreactive for GnRH was calculated as the number in the preoptic area (POA) plus the number in the lateral hypothalamus (LHy). Unless indicated otherwise, data presented are means (± SEM) and *P*-value statistics are the results of student’s t-tests. A superscript letter indicates that data presented are medians (± IQR) and *P*-value statistics are the results of Mann Whitney U tests. AU = arbitrary units.

|  |  | Habitat | | Statistics |  |
| --- | --- | --- | --- | --- | --- |
|  |  | Urban | Desert | *P*-value | |
| **GnRH** | Number of cells in the POA^a^ | 624 (402) | 843.5 (612) | 0.44 | |
|  | Number of cells in the LHy^a^ | 582 (714) | 616 (504) | 0.72 | |
|  | Total number of cells^a^ | 1142 (1226) | 1520 (1232) | 0.50 | |
|  | Cell body area (µm^2^) | 73.2 (± 5.2) | 85.2 (± 3.5) | 0.077 | |
|  | Cell body optical density (AU) | 0.31 (± 0.02) | 0.32 (± 0.03) | 0.75 | |
|  | ME fiber optical density (AU) | 0.20 (± 0.016) | 0.21 (± 0.017) | 0.30 | |
| **GnIH** | Number of cells^a^ | 1316 (1014) | 1322 (928) | 0.72 | |
|  | Cell body area (µm^2^) | 87.9 (± 4.8) | 91.1 (± 9.1) | 0.76 | |
|  | Cell body optical density (AU)^a^ | 0.28 (0.34) | 0.35 (0.15) | 0.57 | |
|  | ME fiber optical density (AU) | 0.104 (±0.015) | 0.094 (± 0.017) | 0.65 | |
| **NPY** | Number of cells^a^ | 348 (178) | 484 (548) | 0.44 | |
|  | ME fiber optical density (AU)^a^ | 0.32 (0.07) | 0.33 (0.09) | 0.96 | |

^a^ data are presented as medians and interquartile range.

**Table S2.** Vernal recrudescence of luteinizing hormone and testosterone secretion, and testicular recrudesence of free-ranging adult male Abert’s towhees, *Melozone aberti*, did not differ between urban and desert populations (*n* = 8 birds per habitat). The *P*-value statistics are the results of Student’s t-tests. Data presented are means (± SEM).

|  | Habitat | | | Statistics |  |
| --- | --- | --- | --- | --- | --- |
|  | Urban | Desert | *P*-value | | |
| Plasma luteinizing hormone (ng/ml) | 4.7 (± 0.6) | 3.6 (± 0.4) | 0.15 | | |
| Paired testis mass (mg) | 415.6 (± 38.7) | 349.5 (± 64.5) | 0.39 | | |
| Seminiferous tubule diameter (µm) | 397.8 (± 23.4) | 328.4 (± 27.0) | 0.07 | | |
| Plasma testosterone (ng/ml) | 7.5 (± 3.4) | 7.6 (± 3.8) | 0.94 | | |

**Table S3.** Plasma luteinizing hormone (LH) and testosterone (T) levels (ng/ml) of free-ranging adult male Abert’s towhees, *Melozone aberti*, bled within 3 min of capture (initial) and again 3 min (LH) and 20 min (T) after (post-injection) an injection of either gonadotropin-releasing hormone-I or saline. Data presented are means (± SEM) and *n* = 8 per group.

|  |  | Habitat | | | |
| --- | --- | --- | --- | --- | --- |
|  |  | Urban | | Desert | |
|  | Treatment | Initial | Post-injection | Initial | Post-injection |
| Plasma luteinizing hormone (ng/ml) | Saline | 4.8 (± 0.7) | 4.0 (± 0.7) | 3.7 (± 0.4) | 3.8 (± 0.4) |
|  | GnRH | 3.5 (± 0.3) | 8.0 (± 1.2) | 3.8 (± 0.5) | 8.0 (± 0.6) |
| Plasma testosterone (ng/ml) | Saline | 5.0 (± 1.2) | 1.8 (± 0.5) | 8.6 (± 2.5) | 2.4 (± 0.5) |
|  | GnRH | 7.4 (± 3.4) | 11.1 (± 1.8) | 7.0 (± 2.5) | 19.2 (± 3.0) |
